# Intensity modulated arc therapy implementation in a three phase adaptive ^18^F-FDG-PET voxel intensity-based planning strategy for head-and-neck cancer

**DOI:** 10.1186/s13014-016-0629-3

**Published:** 2016-04-02

**Authors:** Dieter Berwouts, Luiza Ana Maria Olteanu, Bruno Speleers, Frédéric Duprez, Indira Madani, Tom Vercauteren, Wilfried De Neve, Werner De Gersem

**Affiliations:** Department of Radiotherapy, Ghent University Hospital, De Pintelaan 185, 9000 Ghent, Belgium; Department of Nuclear Medicine, Ghent University Hospital, Ghent, Belgium; Ghent University, Ghent, Belgium; Zürich University Hospital, Zürich, Switzerland

**Keywords:** Adaptive intensity modulated arc therapy, Dose-painting, Intensity modulated radiotherapy, Head-and-neck cancer

## Abstract

**Background:**

This study investigates the implementation of a new intensity modulated arc therapy (IMAT) class solution in comparison to a 6-static beam step-and-shoot intensity modulated radiotherapy (s-IMRT) for three-phase adaptive ^18^F-FDG-PET-voxel-based dose-painting-by-numbers (DPBN) for head-and-neck cancer.

**Methods:**

We developed ^18^F-FDG-PET-voxel intensity-based IMAT employing multiple arcs and compared it to clinically used s-IMRT DPBN. Three IMAT plans using ^18^F-FDG-PET/CT acquired before treatment (phase I), after 8 fractions (phase II) and CT acquired after 18 fractions (phase III) were generated for each of 10 patients treated with 3 s-IMRT plans based on the same image sets. Based on deformable image registration (ABAS, version 0.41, Elekta CMS Software, Maryland Heights, MO), doses of the 3 plans were summed on the pretreatment CT using validated in-house developed software. Dosimetric indices in targets and organs-at-risk (OARs), biologic conformity of treatment plans set at ≤5 %, treatment quality and efficiency were compared between IMAT and s-IMRT for the whole group and for individual patients.

**Results:**

Doses to most organs-at-risk (OARs) were significantly better in IMAT plans, while target levels were similar for both types of plans. On average, IMAT ipsilateral and contralateral parotid mean doses were 14.0 % (*p* = 0.001) and 12.7 % (*p* < 0.001) lower, respectively. Pharyngeal constrictors D_50%_ levels were similar or reduced with up to 54.9 % for IMAT compared to s-IMRT for individual patient cases. IMAT significantly improved biologic conformity by 2.1 % for treatment phases I and II. 3D phantom measurements reported an agreement of ≥95 % for 3 % and 3 mm criteria for both treatment modalities. IMAT delivery time was significantly shortened on average by 41.1 %.

**Conclusions:**

IMAT implementation significantly improved the biologic conformity as compared to s-IMRT in adaptive dose-escalated DPBN treatments. The better OAR sparing and faster delivery highly improved the treatment efficiency.

**Electronic supplementary material:**

The online version of this article (doi:10.1186/s13014-016-0629-3) contains supplementary material, which is available to authorized users.

## Background

Intensity-modulated radiation therapy (IMRT) has become a standard treatment of head-and-neck cancer due to its ability to decrease radiation-induced toxicity [1–3], though the survival rates have not been significantly improved. Since its introduction, different delivery techniques have evolved to make IMRT faster, more precise and flexible. At present, static, dynamic and rotational IMRT are in use demonstrating comparable dose coverage and conformity [4, 5]. Because of a faster delivery, rotational techniques like intensity-modulated arc therapy (IMAT) gained widespread use over recent years. A comparison of different rotational techniques has already been done in literature and it is beyond the scope of this paper [6]. Commercial solutions to perform IMAT are currently available for as well Elekta (Crawley, UK) as Varian (Palo Alto, CA, USA).

In planning studies for head-and-neck cancer, IMAT demonstrated better sparing of organs-at-risk (OARs) without increasing integral dose when compared to static or dynamic IMRT [4–6]. IMAT has the ability to modulate intensities at an infinite number of gantry angles resulting in superior, highly structured dose distributions that are needed for dose painting, i.e., mapping dose to tumor heterogeneity detected by biologic imaging. Up to now, clinical dose-painting by numbers for head-and-neck cancer was based on non-rotational IMRT [7, 8]. The potential of biological image-based IMAT has not been explored yet. We developed an ^18^F-FDG-PET-voxel intensity-based IMAT class solution and investigated its possible implementation in comparison to clinically used adaptive step-and-shoot ^18^F-FDG-PET-voxel intensity-based IMRT (s-IMRT). Herewith we present the results of our study.

## Methods

### Study population

The first 10 head-and-neck cancer patients treated with adaptive ^18^F-FDG-PET-voxel intensity-based IMRT in a randomized phase II dose-escalation clinical trial (NCT01341535) were selected for this study (Table 1). All tumors were biopsy-proven non-metastatic head-and-neck squamous cell carcinomas.

**Table 1:** Patient characteristics

Patient No.	Age (years)	Tumor site	Tumor subsite	TN-stage
1	64	Oropharynx	Tonsil	cT4a pN2b
2	48	Oropharynx	Base of Tongue	cT1 cN2c
3	54	Oropharynx	Tonsil	cT4a cN2c
4	74	Hypopharynx	Aryepiglottic Fold	cT2 cN1
5	40	Hypopharynx	Piriform Sinus	cT1 pN2a
6	53	Larynx	Glottis	cT3 cN0
7	52	Oropharynx	Vallecula	cT1 pN2b
8	54	Oropharynx	Tonsil	cT2 cN2c
9	59	Larynx	Supraglottis	cT2 cN0
10	58	Oropharynx	Vallecula	cT4a cN2c

### Imaging and target definition

All patients were positioned with a five-point thermoplastic mask (Orfit Industries N.V., Belgium), which extended down to the shoulders, during computed-tomography (CT) isocenter simulation and treatment delivery. Planning CT scans of 3 mm slice thickness were acquired before the treatment and after the 8^th^ and 18^th^ fraction. A verification CT was taken at the treatment end. Contrast-enhanced ^18^F-FDG-PET/CT (Philips Medical Systems, Germany) was performed before treatment and after the 8^th^ fraction. ^18^F-FDG-PET-images were acquired with a voxel size of 4 x 4 x 4 mm^3^ as described earlier [9]. Fusion of the planning CT and ^18^F-FDG-PET/CT scans was done on a Pinnacle treatment planning system, version 9.0 (Philips Medical Systems, Andover, MA).

Delineation of the gross tumor volume of the primary tumor (GTV_T_) and pathological lymph nodes (GTV_N_) was done using mutual information of both anatomical and biological imaging. A threshold level of 50 % of SUV_MAX_ (maximal standardised uptake value) was set for ^18^F-FDG-uptake in Pinnacle. Pathologic lymph nodes were delineated separately and noted as the GTV_N1_ and GTV_N2_. The high-risk clinical target volume (CTV_HR_) was created combining the GTV_N_ and a three-dimensional expansion of the GTV_T_ with 1 cm and adjusted to the air cavities and uninvolved bones. 3 mm margin to the CTV_HR_ was used to create the high risk planning target volume (PTV_HR_). Delineation of the elective neck regions according to the guidelines of Gregoire et al. [10] resulted in the CTV of the elective neck (CTV_EN_) and the elective neck PTV (PTV_EN_) after a 3 mm expansion in all directions.

The considered organs-at-risk (OARs) were spinal cord, brainstem, swallowing structures defined as one region-of-interest (superior, medial and inferior pharyngeal constrictor, upper oesophageal sphincter, first 2 cm of the oesophagus and supraglottic larynx), parotids and mandible. Planning OAR volumes (PRVs) were created for the spinal cord and brainstem by three-dimensional expansions of 5 mm and 3 mm, respectively.

Deformable image co-registration (ABAS, version 0.41, Elekta CMS Software, Maryland Heights, MO) was used to propagate the targets and OAR contours from one CT to another in chronological order. All structures were reviewed and edited if necessary by an experienced head-and-neck radiation oncologist.

### Dose prescription and treatment planning

Treatment phases I, II and III consisted of 10 fractions planned on the 1^st^, 2^nd^ and 3^rd^ CT set, respectively. Dose-painting was performed in GTV_T_ and GTV_N_ during the first 20 fractions. The dose range was between 2.2 Gy and 3.1 Gy per fraction in phases I and II. Only a GTV_T_ volume ≤ 1.75 cm^3^ was allowed to receive more than 2.9 Gy per fraction. GTV_N_ was dose-painted in 4 out of 6 patients with N+ disease; in the 2 other patients, which had a pathological lymph node volume ≤ 4 cm^3^, the GTV_N_ median prescription dose was 2.2 Gy per fraction. The total dose range for the GTV_T_ and GTV_N_ was 66-83 Gy.

No dose-painting was performed during the last 10 fractions, where a D_95%_ of 2.0 Gy/fx was prescribed to PTV_HR_. Elective neck was irradiated during fractions 1-20 with a median total dose prescription of 40 Gy to PTV_EN_. GTV_T_ and GTV_N_ biologic conformity was measured by a quality factor (QF), defined as the mean deviation between prescribed and planned dose in each PET/CT voxel [9]. QF was kept below 5 % where possible. Every treatment was planned to a total of 30 fractions and then rescaled to 10 fractions. Maximum doses of 50, 60 and 70 Gy were allowed to < 5 % of the spinal cord (PRV), brainstem (PRV) and mandible, respectively. A maximal dose of less than 45 Gy for the spinal cord, 50 Gy for the brainstem and 27 Gy to < 50 % of the volume of the spared parotids, respectively, were considered clinically acceptable.

The methodology of ^18^F-FDG-PET voxel intensity-based DPBN has been previously discussed [9]. Briefly, a dose is prescribed to the voxels in the dose-painted target volume as a function of signal intensity as follows:

$$ D(I)\kern0.5em =\kern0.5em {D}_{low}\kern17.75em I\kern0.5em \le {I}_{low} $$

$$ D(I)\kern0.5em =\kern0.5em {D}_{low}+\frac{I-{I}_{low}}{I_{high}-{I}_{low}}\left({D}_{high}-{D}_{low}\right)\kern4.75em {I}_{low}\le I\le {I}_{high} $$

$$ D(I)\kern0.5em =\kern0.5em {D}_{high}\kern17em {I}_{high}\le I $$

where the signal intensities I_high_ and I_low_ are determined as 95 % of the maximum ^18^F-FDG-PET intensity and as 25 % of I_high_, respectively. The extension of the discrete PET intensity data to the continuum was implemented using trilinear interpolation for the randomly seeded points in the delineated volumes. Using the PET-intensity to dose relation, the dose prescription was on a point-by-point base.

All treatment plans were created for an Elekta linac (Crawley, UK) equipped with a standard multileaf collimator with 40 leaf pairs, capable of delivering s-IMRT and IMAT with variable dose rate, gantry and collimator rotation speed. In-house developed software using an anatomy- and ^18^F-FDG-PET-voxel intensity-based segmentation tool (ABST, BBST) followed by leaf position and monitor unit (MU) optimization was used for treatment planning [11, 12].

s-IMRT plans consisted of six non-opposing coplanar 6 MV beams with gantry angles of 45°, 75°, 165°, 195°, 285° and 315°. The IMAT class solution was made of 6 MV arcs collimated around PTV_EN_ (gantry angle from -176° to 176°) and PTV_HR_ (144° to -144°) with control points (CPs) defined every 8°. The only constraints were on the physical abilities of the linear accelerator to deliver the treatment (maximum gantry speed, maximum collimator rotation speed, maximum leaf speed, minimum dose rate), and a minimum distance constraint of 1 cm for opposite and diagonally-opposite leaves of the MLC. ABST [11] was used to create the starting set of CPs, resulting in multiple initial arcs, avoiding both parotids, the swallowing structures and the PRV of the spinal cord. The CPs were optimized as described previously [12]. ABST generates beam segments with leaf and jaw positions based on a beams-eye-view projection of selected PTVs and OARs. BBST additionally takes into account PET-intensities to create initial beam segments shapes [9]. For a faster delivery, the parts of the arcs with a contribution of less than 2 MUs were eliminated during the optimization leading to the split of the arcs in sub-arcs. A CP refinement was performed by interpolating and generating additional CPs within the arcs, followed by MU and leaf position optimization. This CP refinement limited MU differences, gantry and collimator angle differences, leaf and jaw position movements between CPs and was applied to reach the accuracy constraints used in the treatment verification. After the final optimization, the remaining arcs were linked together in one beam according to the shortest possible delivery time. All dose computations were done in Pinnacle with a collapsed cone convolution/superposition calculation algorithm.

### Dose reporting and statistical analysis

Doses of the 3 treatment plans were summed on the pretreatment CT using in-house developed software [13] based on the deformable CT image registrations made with the ABAS software. The reporting of the region-of-interest (ROI) dose levels was done on the summed doses.

To assess the risk of inducing secondary malignancies, the integral dose was calculated in the patient volume as follows:

$$ \mathrm{ID}\kern0.5em =\kern0.5em {\mathrm{D}}_{\mathrm{mean}}\cdot \mathrm{V}\cdot \uprho $$

where D_mean_ is the mean dose, V is the volume and ρ the tissue density, which was considered to be 1 g/cm^3^.

Statistical tests of dosimetric, biologic conformity, treatment verification and quality (MUs and delivery time) differences between s-IMRT and IMAT were done using a two-sided Wilcoxon matched-pair signed rank test with SPSS software version 20.0 (SPSS Inc., Chicago, IL). Differences were considered statistically significant for *p*-values <0.05.

### Treatment verification

The delivered dose distributions of all IMAT and s-IMRT treatment plans were verified with the 3D dosimetry system Delta^4^ (Scandidos, Uppsala, Sweden). The Delta^4^ phantom has 1069 p-type disc-shaped Silicon diodes with a diameter of 1 mm and axial size 0.05 mm, in a central region (6x6 cm) spaced per 5 mm, outside the central region spaced per 10 mm. Global gamma indices [14] were determined in the Delta^4^ control software for the criteria of 3 % dose difference and 3 mm distance-to-agreement, the normalization dose being the prescribed dose.

The delivery treatment time was also recorded from the start of the first beam till the end of the last beam.

## Results

### Dosimetrical and biological conformity results

Population average dose-volume parameters of targets and OARs for both strategies are shown in Table 2. Most of the differences between s-IMRT and IMAT for target and OAR dose levels were significant (Table 2). Mean V_27Gy_ of ipsi- and contralateral parotids were improved by 16.4 % (*p* = 0.007) and 17.5 % (*p* = 0.003) in the IMAT plans, respectively. For the volume of interest that comprised the pharyngeal constrictor muscles (PC) and the one that combined the swallowing structures (SS), both D_50%_ and D_98%_ levels were significantly improved in the IMAT plans, while D_2%_ did not show on average any important differences.

**Table 2:** Population average dose levels for s-IMRT and IMAT treatments

Target/Organ-at-risk	s-IMRT (Gy)	IMAT (Gy)	*p*-value
GTV_T_			
D_2%_	80.4 (76.4 - 83.7)	81.0 (77.2 - 85.1)	0.175
D_98%_	67.3 (63.8 – 71.0)	68.4 (64.8 - 73.6)	**0.009**
GTV_N_			
D_2%_	73.3 (66.4 - 79.3)	74.8 (67.2 - 81.0)	0.014*
D_98%_	64.8 (62.5 - 69.9)	66.3 (63.1 - 72.8)	0.043*
PTV_HR_			
D_2%_	76.9 (74.5 - 79.6)	77.9 (75.8 - 79.3)	**0.013**
D_98%_	57.5 (54.3 - 59.4)	56.9 (52.5 - 58.7)	0.160
PTV_EN_			
D_2%_	66.0 (61.1 - 73.3)	67.0 (62.0 – 76.0)	**0.005**
D_98%_	32.2 (21.2 - 42.2)	32.7 (20.2 - 40.8)	0.452
CTV_HR_			
D_2%_	78.1 (75.6 - 81.2)	79.1 (76.6 - 81.3)	**0.023**
D_98%_	60.9 (59.6 - 61.6)	60.3 (58.9 - 61.5)	**0.025**
CTV_EN_			
D_2%_	66.5 (59.7 - 75.9)	67.8 (61.7 - 77.9)	**0.001**
D_98%_	38.9 (35.2 - 44.5)	38.6 (34.1 - 42.2)	0.222
Spinal cord PRV			
D_5%_	32.4 (29.5 - 34.7)	28.4 (23 - 35.3)	**0.003**
D_50%_	21.4 (3.5 - 28.5)	13.6 (1.8 - 21.5)	**0.001**
Brainstem PRV			
D_5%_	20.5 (12.4 - 28.4)	14.2 (6.1 - 23.6)	**<0.001**
D_50%_	2.3 (1.4 - 3.1)	2.1 (1.1 - 3.4)	**0.019**
Ipsilateral parotid			
V_27Gy_	43.8 (34.8 - 52.5)	36.6 (24.6 - 48.1)	**0.007**
D_mean_	25.8 (18.7 - 31.8)	22.2 (14.9 - 29.4)	**0.001**
Contralateral parotid			
V_27Gy_	40.0 (27.6 - 50.1)	33.0 (13.2 - 47.4)	**0.003**
D_mean_	24.4 (16.9 - 30.6)	21.3 (12.5 - 27.5)	**<0.001**
PC			
D_2%_	67.3 (63.9 - 73.3)	67.6 (63.5 – 74.0)	0.469
D_50%_	57.0 (49.1 – 62.0)	53.4 (38.2 - 60.8)	**0.021**
D_98%_	36.3 (19.4 - 51.3)	25.1 (11.1 - 43.7)	**<0.001**
SS			
D_2%_	68.0 (61.0 - 77.6)	68.0 (60.8 - 76.6)	0.903
D_50%_	53.5 (40.5 - 63.8)	47.4 (32.5 - 63.2)	**0.003**
D_98%_	34.3 (24.1 - 40.3)	23.3 (12.4 - 31.2)	**<0.001**
Mandible			
D_2%_	53.6 (34.4 - 68.1)	53.6 (35.0 - 66.9)	0.923

The dose distributions of the 3 treatment phases were summed on the pretreatment CT. Reporting is done on manually delineated targets and organs-at-risk. Statistically significant differences are shown in bold

Abbreviations: *s-IMRT* step-and-shoot IMRT, *IMAT* intensity modulated arc therapy, *GTV*_*T*_ gross tumor volume of the primary tumor, *GTV*_*N*_ GTV of the metastatic lymph nodes, *CTV*_*HR*_ high-risk clinical target volume, *PTV*_*HR*_ high-risk planning target volume, *CTV*_*EN*_ elective neck CTV, *PTV*_*EN*_ elective neck PTV, *PRV* planning organ-at-risk volume, *SS* swallowing structures include the superior, middle and inferior pharyngeal constrictor muscles, upper esophageal sphincter, supraglottic larynx and upper 2 cm of the cervical esophagus, *PC* pharyngeal constrictors include the superior, middle and inferior pharyngeal constrictor muscles, *D*_*x%*_ dose received by x% of the volume, *V*_*27Gy*_ % of the volume that receives at least 27 Gy

*Of 10 patients, 6 had metastatic lymph nodes

**Fig. 1:**
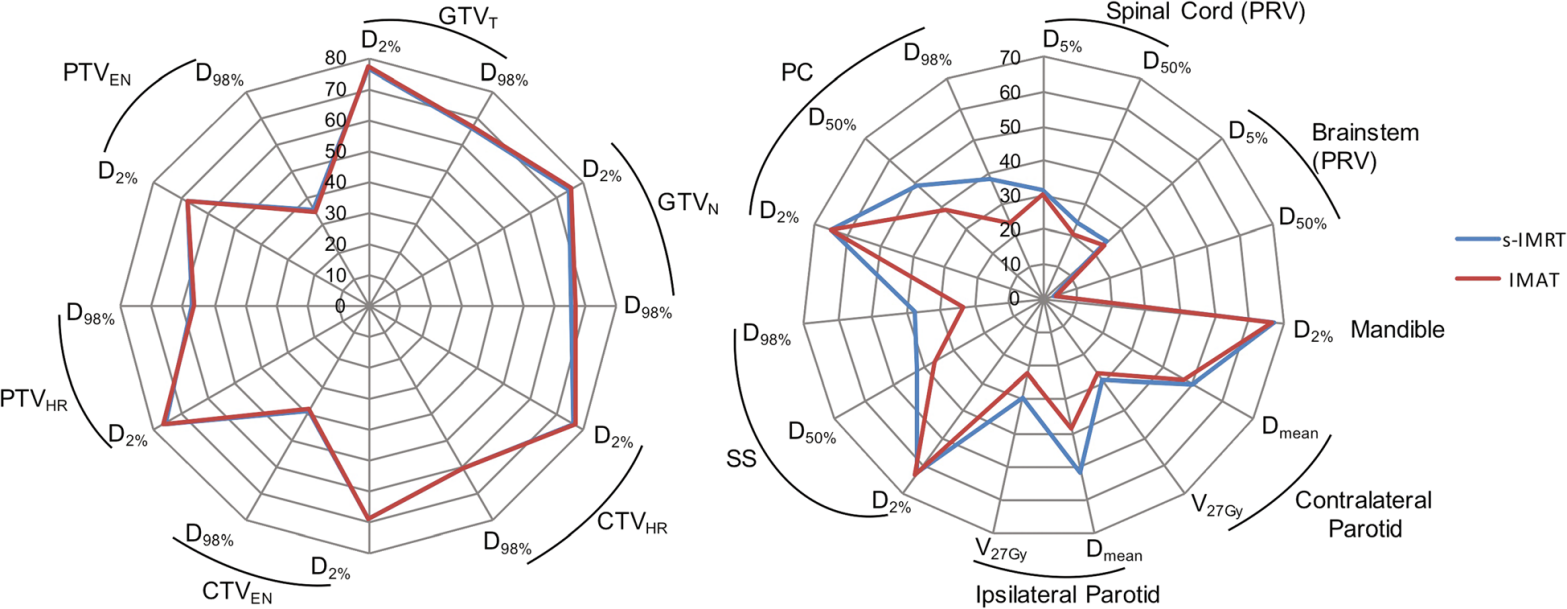
Radar charts of dose/volume levels comparing s-IMRT and IMAT plans summed on the pretreatment CT for a patient with a cT4a pN2 cM0 oropharynx cancer. The areas are formed by connecting the values belonging to one of the two treatment strategies. *Abbreviations:* s-IMRT = step-and-shoot IMRT; IMAT = intensity modulated arc therapy; GTV_T_ = gross tumor volume of the primary tumor; GTV_N_ = GTV of the metastatic lymph nodes; CTV_HR_ = high risk clinical target volume; PTV_HR_ = high risk planning target volume; CTV_EN_ = elective neck CTV; PTV_EN_ = elective neck PTV; PRV = planning organ-at-risk volume; SS = swallowing structures – includes superior pharyngeal constrictor, middle pharyngeal constrictor, inferior pharyngeal constrictor, upper esophageal sphincter, supraglottic larynx and upper 2 cm of the esophagus; PC = pharyngeal constrictors – includes superior pharyngeal constrictor, middle pharyngeal constrictor and inferior pharyngeal constrictor; D_x%_ = dose received by x% of the volume; V_27Gy_ = % of the volume that receives at least 27 Gy

Analysis of each summed dose distribution separately revealed larger differences for some cases in comparison with average data. Additional file 1: Figure S1 showed similar or highly reduced D_50%_ and D_98%_ levels of PC and SS with up to 54.9 % for IMAT compared to IMRT. D_2%_ differences of the same structures varied from -2.8 % to 3.6 %. For a cT4a pN2 cM0 oropharynx cancer case the results were plotted in Fig. 1. IMAT ipsilateral and contralateral parotid mean dose was lowered by 24.9 % and 5.3 %, respectively, while V_27_ was also improved by 24.9 % and 6.7 %, respectively. Additional file 2: Figure S2 provides for the same patient a visual image of how IMAT isodoses better spare the parotids on every treatment phase, except for the contralateral parotid on the third treatment phase. The s-IMRT median dose of the swallowing structures was 22.7 % and 12.3 % higher for the PC and SS structures.

GTV_T_ quality factors (QF) were significantly better for the IMAT-plans (*p* < 0.001 for both DPBN-phases) with a maximum difference from IMRT factors of -2.1 % in phase I and II (Table 3). When the QF values of GTV_T_ and GTV_N_ were considered as one group, a Wilcoxon test also showed significantly (*p* < 0.001) lower values for IMAT.

**Table 3:** Quality factors (%) of s-IMRT and IMAT dose-painting by numbers plans of the first two treatment phases

	Phase I		Phase II	
	sIMRT	IMAT	sIMRT	IMAT
Patient 1				
GTV_T_	5.1	3.9	4.3	2.8
GTV_N1_	1.8	1.5	4.6	1.4
GTV_N2_	2.8	2.0		
Patient 2				
GTV_T_	5.0	3.6	4.0	3.2
GTV_N1_	3.1	2.9	5.3	3.8
Patient 3				
GTV_T_	4.3	2.8	3.6	2.2
Patient 4				
GTV_T_	4.0	4.5	2.6	3.4
Patient 5				
GTV_T_	5.1	3.5	4.5	3.1
Patient 6				
GTV_T_	4.6	2.9	3.2	2.8
Patient 7				
GTV_T_	3.8	4.1	3.4	2.9
Patient 8				
GTV_T_	4.0	2.3	4.9	2.8
GTV_N1_	2.2	2.5		
GTV_N2_	2.6	2.7		
Patient 9				
GTV_T_	3.5	1.9	2.8	1.4
Patient 10				
GTV_T_	5.1	4.1	5.1	4.2
GTV_N1_	3.9	3.4	5.1	2.4

Dose painting inside GTV_N_ was done only for the cases where the PET signal was high enough

Abbreviations: *s-IMRT* step-and-shoot IMRT, *IMAT* intensity modulated arc therapy, *GTV*_*T*_ gross tumor volume of the primary tumor, *GTV*_*Nx*_ metastatic ^18^F-FDG-PET-positive lymph node

The integral dose inside the patient was lower for IMAT in 7 patients with a maximum difference of 14.4 % (Table 4). For 2 cases, IMAT integral dose was with 2.8 and 3.7 % higher, while for one case it was similar.

**Table 4:** Integral Dose (J) calculated on the pretreatment CT scans inside the patient volume

Patient No.	s-IMRT	IMAT	Δ%
1	243.1	238.4	-1.9
2	274.4	274.3	0.0
3	115.3	109.1	-5.4
4	125.1	123.6	-1.2
5	135.3	129.1	-4.6
6	120.5	124.9	3.7
7	130.2	128.9	-1.0
8	150.1	128.6	-14.4
9	104.4	101.6	-2.7
10	160.1	164.6	2.8

Abbreviations: *s-IMRT* step-and-shoot IMRT, *IMAT* intensity modulated arc therapy

### Delivery results

The data on dosimetric verification of treatment plans, number of MUs and delivery time are presented in Table 5. The number of MUs was significantly higher for IMAT than for s-IMRT plans. All treatments were delivered on the Elekta linacs while measuring with the Delta^4^ system. Mean percentages of the points with gamma index >1 were 99.7 ± 0.6 % versus 98.7 ± 1.3 % for s-IMRT and IMAT, respectively. On average, s-IMRT treatment times of phases I, II and III were 6:52, 6:39 and 5:00 min, respectively. IMAT delivery was significantly shorter: 4:13, 3:44 and 2:56 min, respectively.

**Table 5:** Delivery analysis of each treatment phase: number of monitor units (MUs), treatment time (minutes:seconds) - registered from the beginning of the first beam till the end of the last beam - and the percentage of the measurement points with γ < 1 - which compares Delta^4^ measurements and Pinnacle dose calculations

Patient	Phase	Number of MUs		Treatment time		% of points with γ < 1	
		s-IMRT	IMAT	s-IMRT	IMAT	s-IMRT	IMAT
1	I	609	792	8:13	5:15	100.0	98.4
	II	515	671	8:03	3:49	100.0	96.8
	III	599	777	6:05	3:41	100.0	98.5
2	I	646	742	9:00	4:27	100.0	98.6
	II	646	803	8:13	3:30	100.0	97.5
	III	448	616	5:50	3:35	99.4	98.9
3	I	415	725	6:11	3:56	100.0	97.8
	II	436	734	6:30	3:15	100.0	96.1
	III	310	877	5:00	4:12	97.3	94.3
4	I	384	737	5:50	3:48	100.0	98.8
	II	409	671	5:55	4:12	99.6	98.6
	III	283	575	5:02	3:05	99.4	98.7
5	I	438	719	5:50	3:47	100.0	99.7
	II	417	751	6:12	3:12	100.0	97.4
	III	235	226	4:24	1:27	100.0	100.0
6	I	520	809	6:13	3:14	100.0	99.8
	II	466	748	5:55	3:23	100.0	98.4
	III	263	424	4:22	2:35	100.0	100.0
7	I	538	695	6:10	4:20	100.0	99.9
	II	591	738	6:30	3:49	100.0	100.0
	III	263	664	5:00	3:05	99.5	99.4
8	I	689	672	9:00	3:36	99.7	99.0
	II	437	595	6:32	4:06	100.0	100.0
	III	368	740	4:15	3:54	99.8	98.2
9	I	450	708	5:26	4:51	100.0	99.2
	II	464	674	5:41	3:34	99.9	99.7
	III	315	322	4:46	1:56	98.4	100.0
10	I	545	746	6:47	5:03	100.0	100.0
	II	562	759	7:07	4:37	99.8	98.6
	III	336	270	5:25	1:52	99.4	99.6
*p*-value		< .0001		< .0001		< .0001	

Two-sided Wilcoxon matched-pair signed rank test *p*-values are given on the last row

Abbreviations: *s-IMRT* step-and-shoot IMRT, *IMAT* intensity modulated arc therapy

## Discussion

In this study we demonstrated the feasibility of a new ^18^F-FDG-PET-voxel intensity-based IMAT class solution in our adaptive dose-painting strategy. DPBN imposes heavy demands to treatment planning and delivery technology including high dose gradients and high degree of fluence modulation. Until now ^18^F-FDG-PET-voxel intensity-based s-IMRT has been used in DPBN trials for head-and-neck cancer [7, 8]. Probably due to limited modulation of s-IMRT in comparison to IMAT, biologic conformity of s-IMRT-based DPBN plans was not systematic. Severe toxicity was also experienced with DPBN-based dose escalation s-IMRT treatments [7] e.g. mucosal ulcers and dysphagia. Preliminary data from our clinical trials suggests that severe toxicity was correlated with dose-escalation and with smoking and alcohol abuse during and after treatment. There was no indication that severe toxicity could be caused by IMRT or the dose painting concept itself. The search to decrease the toxicity of dose-escalated treatments by reducing the OAR doses lead to the development of ^18^F-FDG-PET-voxel intensity-based IMAT.

We proposed a method using multiple partial arcs that would ensure higher flexibility and better conformity in dose distributions. In IMAT plans, the dose-painting quality factor evaluating biologic conformity of treatment plans showed significantly better values than for s-IMRT plans. Although most of the differences in D_2%_ and D_98%_ for the target structures were significant, they were not clinically relevant on both individual and average patient data.

Previous studies showed that in complex-shaped targets as head-and-neck cancer using a single arc was not sufficient to reach the quality of IMRT plans [15]. Most publications report similar or slightly better IMAT plans (dose coverage and homogeneity in targets) in comparison with dynamic IMRT or static IMRT at conventional dose prescription, when double or triple full arcs were used [4, 5, 15–20].

IMAT has the potential to decrease doses to OARs [4, 5, 15–21] that becomes crucially important in dose-escalation treatment protocols. In s-IMRT plans we usually sacrifice the ipsilateral parotid, if the tumor or metastatic lymph node is at the level of the gland. A previous study [22] showed that adapting treatment to anatomic changes in the glands could lower doses even in the ipsilateral parotid. The current study results demonstrate that IMAT could further spare both parotids by significantly reducing D_mean_ (by 14.0 % and 12.7 % for the ipsilateral and contralateral parotid, respectively) and V_27Gy_ (by 16.4 % and 17.5 % for the ipsilateral and contralateral parotid, respectively) as compared to s-IMRT, both treatments being adaptive. Vanetti et al*.* [5] obtained a significant reduction of parotid D_mean_ using two full arcs against dynamic IMRT by 14.0 % and 13.5 % for the ipsilateral and contralateral parotid, respectively. Other studies employing double or triple full arcs demonstrated similar contributions to parotid D_mean_ by IMAT and IMRT [15, 16, 19]. With IMAT we could also better spare other OARs - the spinal cord, brainstem, pharyngeal constrictor muscles and swallowing structures - except the mandible (Table 2) a finding in agreement with Vanetti et al. [5]. Reduction in doses to OARs was even more evident in individual patients (Additional file 1: Figure S1 and Additional file 2: Figure S2).

Most retrospective [4, 5, 15–20] and prospective [21] IMAT-IMRT comparisons report a lower number of MUs for the arc therapy plans, although some report higher MUs [16, 26]. Our IMAT plans had on average higher MUs than IMRT plans, which might be of less concern due to the following reasons. The integral dose inside the patient (Table 4) showed that for IMAT plans the theoretical risk of developing secondary malignancies was less or similar to the s-IMRT plans. By delivering more dose to the surrounding tissues, based on the linear-non-threshold-model, an increase in secondary neoplasm can be expected [23]. Furthermore, the latest commercially available MLC devices are characterized with very low leakage and hence the overall patient exposure to low doses is highly reduced [24–26]. The linac head and MLC leakage is even further reduced in the case of flattening filter free linacs [27].

Our IMAT plan measurements showed that a discrete dose calculation per 8° was not always a good approximation of the arc delivery (data not shown). There are two reasons likely to cause the lower gamma index percentages for the IMAT QA: one is the discretization (to a limited number of gantry angles) used in the dose computation, the second is the higher number of Monitor Units (MU) for the IMAT plans together with smaller fields. By CP refinement and further optimization, gamma percentages higher than 94.3 % could be achieved. The single arc plans of Bertelsen et al*.* [18] gave slightly better average percentages for gamma < 1 (99.6 ± 0.5 %) as compared to the multiple partial arc plans of the present study (98.7 ± 1.3 %). Korreman et al*.* [28] got 89.6 %, 88.5 % and 92.2 % for double arc plans corresponding to 3, 7 and 11 dose-painting-by-contours prescribed levels for one individual case. The reliability of Delta^4^ phantom measurements for IMRT and IMAT was studied by Bedford et al. [29]. We would like to point out that the spacing of 0.5 and 1 cm between the Delta^4^ array detectors was rather limited for the high dose gradients of DPBN plans.

Rotational treatment shortens delivery time thus improving comfort for the patient and reducing risk of patient movement during treatment, which cannot be neglected [30]. By eliminating parts of the arcs with very low contribution and linking them in one arc, IMAT treatment delivery time became in the range 1.3 to 5.2 min, which despite dose escalation, was comparable or even faster than published data on single, double or triple full arc plans using conventional prescription doses to targets [5, 15–20].

## Conclusions

IMAT implementation in an adaptive dose-escalation biological image-guided treatment strategy lead to significantly better biological quality factors in comparison to s-IMRT. The method was superior in reducing dose to OARs, biologic conformity and treatment efficacy. IMAT treatment delivery was significantly faster than s-IMRT and the multiple partial arc class solution made it one of the fastest reported in literature. Hence more patients can be treated per day with more comfort and less intra-fraction movements.

### Ethics approval and consent to participate

The data used in this manuscript are part of the study registerend on clinicaltrials.gov under NCT01341535. This study was approved by the Ethics Committe of Ghent University Hospital.

## Additional files

Additional file 1: Figure S1. Individual patient IMAT and s-IMRT dose levels (D_2%,_ D_50%,_ D_98%_) for the volume of interest that comprises the pharyngeal constrictor muscles (PC) and the one that combines the swallowing structures (SS). Each graph presents the 10 individual patient values of one volume of interest dose level for the total summed dose distribution on the pretreatment CT. X and y axes show the s-IMRT and IMAT dose values (Gy). The marker position above or below the identity line (dotted) correspond to a higher or lower IMAT dose level value in comparison with s-IMRT, respectively. *Abbreviations:* s-IMRT = step-and-shoot IMRT; IMAT = intensity modulated arc therapy; PC = pharyngeal constrictor – includes superior pharyngeal constrictor, middle pharyngeal constrictor and inferior pharyngeal constrictor; SS = swallowing structures – includes superior pharyngeal constrictor, middle pharyngeal constrictor, inferior pharyngeal constrictor, upper esophageal sphincter, supraglottic larynx and upper 2 cm of the esophagus; D_x%_ = dose received by x% of the volume. (TIF 792 kb) Additional file 2: Figure S2. s-IMRT (first row) and IMAT (second row) dose distributions for the 3 treatment phases of a patient with a cT4a pN2 cM0 oropharynx cancer. Isodoses are displayed on the CT transverse images. For phases I and II the contrast-enhanced ^18^F-FDG-PET image set is superposed on the CT. The regions of interest contours are drawn as follows: GTV_T_ and GTV_N_ in red, CTV_HR_ in purple, PTV_HR_ in blue and the parotids in green (colorwash). *Abbreviations:* s-IMRT = step-and-shoot IMRT; IMAT = intensity modulated arc therapy; GTV_T_ = gross tumor volume of the primary tumor; GTV_N_ = GTV of the metastatic lymph nodes; CTV_HR_ = high risk clinical target volume; PTV_HR_ = high risk planning target volume. (TIF 1747 kb)

## Abbreviations

^18^F-FDG-PET: 2-deoxy-2-(^18^F)fluoro-D-glucose positron emitting tomography
ABAS: Elekta’s atlas-based autosegmentation
ABST: anatomy-based segmentation tool
BBST: biology-based segmentation tool
CPs: control points
CT: computed tomography
CTV_HR_: high risk clinical target volume
CTV_EN_: elective neck clinical target volume
D_mean_: mean dose
DPBN: dose-painting-by-numbers
D_x%_: dose received by x % of the volume
GTV_Nx_: gross tumor volume of pathological lymph node(s)
GTV_T_: gross tumor volume of the primary tumor
Gy: gray
IMAT: intensity-modulated arc therapy
MU: monitor unit
MV: megavolt
OAR: organ-at-risk
PC: pharyngeal constrictor
PRV: planning risk volume
PTV_EN_: elective neck planning target volume
PTV_HR_: high risk planning target volume
QF: quality factor
ROI: region of interest
s-IMRT: step-and-shoot intensity modulated radiotherapy
SPSS: IBM statistical package for the social sciences
SS: swallowing structures
SUV_MAX_: maximal standardised uptake value
V_xGy_: volume receiving x Gy
