# Supplementary figures and images for: Intensity modulated arc therapy implementation in a three phase adaptive 18F-FDG-PET voxel intensity-based planning strategy for head-and-neck cancer
Source: Radiat Oncol. 2016 Apr 2;11:52. doi: 10.1186/s13014-016-0629-3 (PMC4818905; doi:10.1186/s13014-016-0629-3)

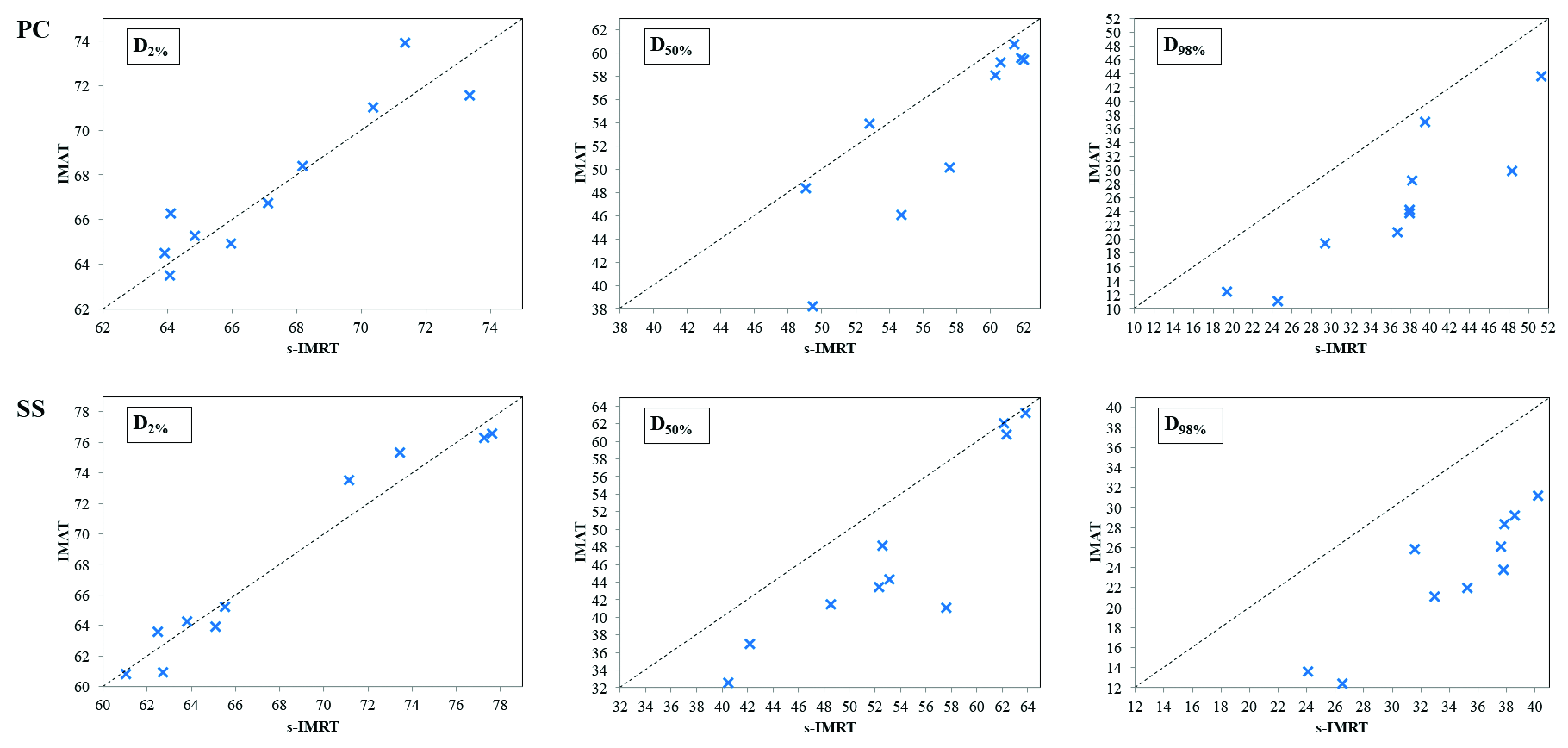

Supplement: Additional file 1: Figure S1. — Individual patient IMAT and s-IMRT dose levels (D2%, D50%, D98%) for the volume of interest that comprises the pharyngeal constrictor muscles (PC) and the one that combines the swallowing structures (SS). Each graph presents the 10 individual patient values of one volume of interest dose level for the total summed dose distribution on the pretreatment CT. X and y axes show the s-IMRT and IMAT dose values (Gy). The marker position above or below the identity line (dotted) correspond to a higher or lower IMAT dose level value in comparison with s-IMRT, respectively. Abbreviations: s-IMRT = step-and-shoot IMRT; IMAT = intensity modulated arc therapy; PC = pharyngeal constrictor – includes superior pharyngeal constrictor, middle pharyngeal constrictor and inferior pharyngeal constrictor; SS = swallowing structures – includes superior pharyngeal constrictor, middle pharyngeal constrictor, inferior pharyngeal constrictor, upper esophageal sphincter, supraglottic larynx and upper 2 cm of the esophagus; Dx% = dose received by x% of the volume. (TIF 792 kb) [file 13014_2016_629_MOESM1_ESM.tif]

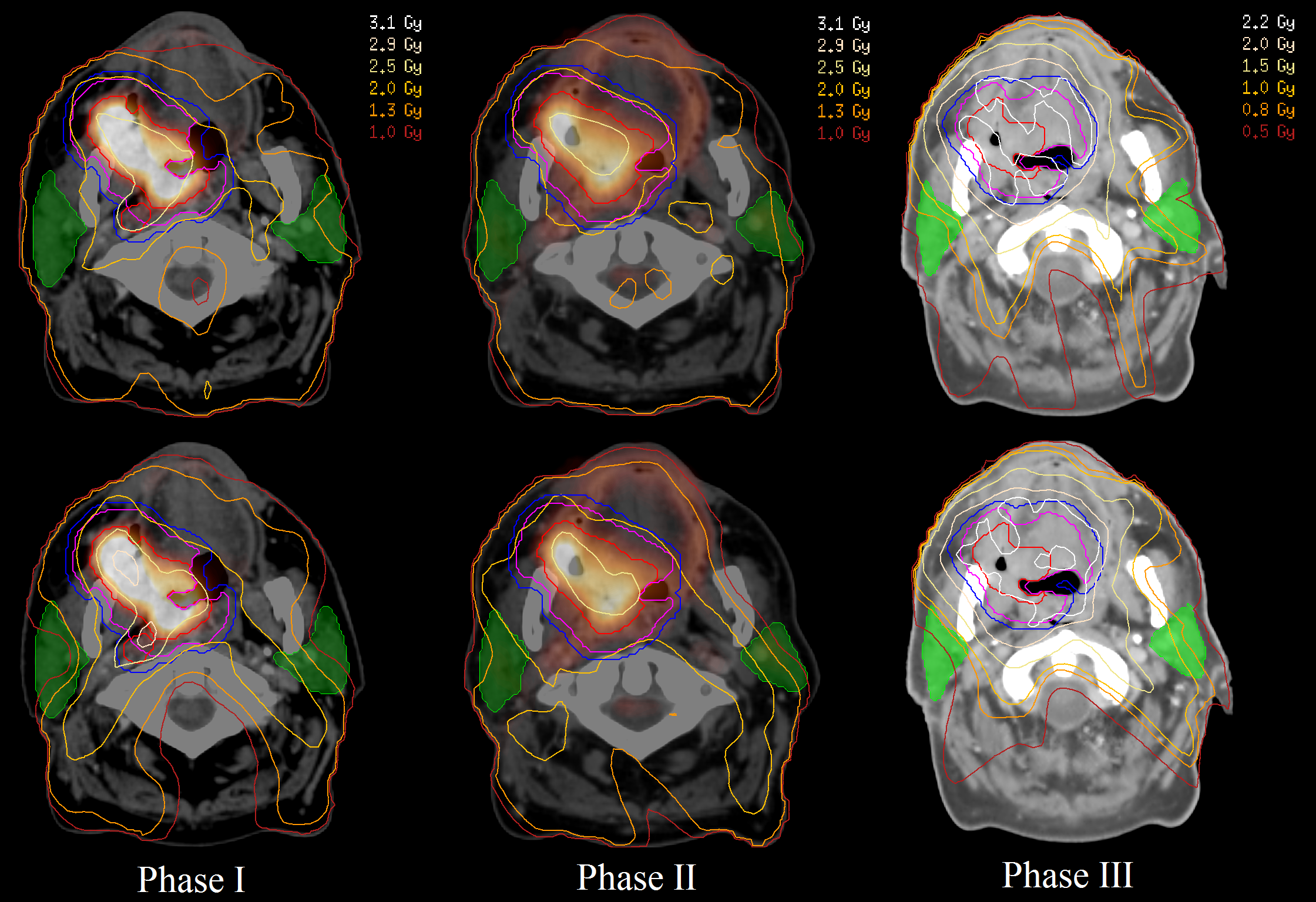

Supplement: Additional file 2: Figure S2. — s-IMRT (first row) and IMAT (second row) dose distributions for the 3 treatment phases of a patient with a cT4a pN2 cM0 oropharynx cancer. Isodoses are displayed on the CT transverse images. For phases I and II the contrast-enhanced 18F-FDG-PET image set is superposed on the CT. The regions of interest contours are drawn as follows: GTVT and GTVN in red, CTVHR in purple, PTVHR in blue and the parotids in green (colorwash). Abbreviations: s-IMRT = step-and-shoot IMRT; IMAT = intensity modulated arc therapy; GTVT = gross tumor volume of the primary tumor; GTVN = GTV of the metastatic lymph nodes; CTVHR = high risk clinical target volume; PTVHR = high risk planning target volume. (TIF 1747 kb) [file 13014_2016_629_MOESM2_ESM.tif]
